# Association of high *Plasmodium falciparum* parasite densities with polyclonal microscopic infections in asymptomatic children from Toubacouta, Senegal

**DOI:** 10.1186/s12936-019-2684-3

**Published:** 2019-02-21

**Authors:** Babacar Diouf, Fode Diop, Yakhya Dieye, Cheikh Loucoubar, Ibrahima Dia, Joseph Faye, Mbacké Sembène, Ronald Perraut, Makhtar Niang, Aïssatou Toure-Balde

**Affiliations:** 1 Immunology Unit, Institut Pasteur Dakar, 36 Avenue Pasteur, BP 220, Dakar, Senegal; 2 Virology Unit, Institut Pasteur Dakar, 36 Avenue Pasteur, BP 220, Dakar, Senegal; 3 Epidemiology of Infectious Diseases Unit, Institut Pasteur Dakar, 36 Avenue Pasteur, BP 220, Dakar, Senegal; 4 Entomology Unit, Institut Pasteur Dakar, 36 Avenue Pasteur, BP 220, Dakar, Senegal; 5 Faculté des Sciences et Techniques, Université Cheikh Anta Diop, P. O. Box 5005, Dakar, Senegal

**Keywords:** *Plasmodium falciparum*, Polymorphism, *msp*-*1*, *msp*-*2*, Asymptomatic

## Abstract

**Background:**

Malaria is a leading cause of mortality and morbidity in tropical countries, especially in sub-Saharan Africa. In Senegal, a control plan implemented in the beginning of the 2000s has enabled a substantial reduction of mortality and morbidity due to malaria. However, eradication of malaria requires a vaccine that protects against *Plasmodium falciparum* the deadliest species of the parasite that causes this disease. *Plasmodium falciparum* is characterized by an extensive genetic diversity that makes vaccine development challenging. In this study, the diversity of *P. falciparum* isolates was analysed from asymptomatic children residing in the district of Toubacouta, Senegal.

**Methods:**

A nested PCR approach was used to perform genotyping of the *msp*-*1* and *msp*-*2* loci in samples from 87 asymptomatic children infected with *P. falciparum*, collected during a cross sectional survey in November and December 2010. Parasite densities in blood samples were determined by microscopic examination and statistical analyses were used to identify association of parasite genotype and parasitaemia.

**Results:**

Genotyping was successful in 84/87 and 82/87 samples for *msp*-*1* and *msp*-*2*, respectively. A strong genetic diversity was found with a total of 15 and 21 different alleles identified for *msp*-*1* and *msp*-*2*, respectively. RO33 was the most frequent allelic family of *msp*-*1* followed by MAD20, then by K1. Regarding *msp*-*2* allelic families, 3D7 was more common than FC27. Multiple infections were predominant, since 69% and 89% of the samples genotyped for *msp*-*1* and *msp*-*2* showed more than one clone of *P. falciparum* with complexity of infection (COI) of 2.5 and 4.7, respectively. Expected heterozygosity (H_E_) was 0.57 and 0.55 for *msp*-*1* and *msp*-*2*, respectively. Interestingly, polyclonal infections were significantly associated with higher parasitaemia.

**Conclusions:**

The strong genetic diversity of *P. falciparum* clones and the association of polyclonal infection with high parasitaemia call for a multi-allelic approach in the design of vaccine candidates for efficient malaria eradication.

**Electronic supplementary material:**

The online version of this article (10.1186/s12936-019-2684-3) contains supplementary material, which is available to authorized users.

## Background

Malaria is a leading cause of mortality and morbidity in tropical countries. In 2016, more than 212 million of clinical cases and 429,000 deaths were reported worldwide. Sub-Saharan African countries support 92% of the global malaria burden, with children younger than 5 years, pregnant women, and non-immune visitors to endemic areas being people the most at risk of developing severe or fatal malaria [1]. In Senegal, 492,253 clinical cases and 526 deaths were reported in 2015. These figures represent a 12-times reduction compared to those from 2001, a substantial decrease that highlights the success of the malaria control programme that was implemented in Senegal in the beginning of the 2000s [2]. This malaria control programme was based on several approaches including widespread use of insecticide-treated mosquito nets (ITNs), rapid diagnostic testing (RDTs), treatment with artemisinin-based combination therapy (ACT), indoor residual spraying of insecticides (IRS), intermittent preventive treatment in pregnancy (IPTp) and seasonal malaria chemoprevention (SMC) in children under 10 years of age with sulfadoxine–pyrimethamine plus amodiaquine in areas of high seasonal malaria transmission [1]. However, despite this undisputable success, multiple factors can threaten malaria control efforts and compromise elimination including the emergence of drug-resistant parasite strains [3], insecticide-resistant mosquito vectors [4] as well as the weakness of the healthcare systems since malaria endemic countries are among the poorest in the world [5].

Achieving malaria elimination requires the development of an effective vaccine, especially against *Plasmodium falciparum,* which is the deadliest of the five human malaria parasites. A major obstacle toward this goal is the extensive genetic diversity of natural parasite populations that complicate the design of an effective vaccine against all *P. falciparum* strains. The difficulty related to *P. falciparum* genetic diversity has been clearly illustrated in clinical trials that tested different vaccine candidates. For example, the malaria vaccine combination B, designed as a three-component blood-stage product targeting the merozoite surface proteins MSP-1 (K1 parasite line) and MSP-2 (3D7), and the ring-infected erythrocyte surface antigen (RESA) was efficient against homologous parasites harboring the 3D7 allelic family of MSP-2, but failed to protect against those containing the FC27 allelic family, and was even associated with an increased rate of morbidity [6]. More recently, the malaria vaccine FMP2.1/AS02 (A), a recombinant protein based on the apical membrane antigen 1 (AMA1) from the 3D7 strain of *P. falciparum* did not provide significant protection against clinical malaria, but showed a strain-specific efficacy [7]. Moreover, a phase III clinical trial with RTS,S, the most advanced malaria vaccine candidate, showed a greater activity against parasites carrying the matching allele of the circumsporozoite protein than against other strains. In this last trial, less than 10% of the 5–17 months old children who were infected harbored parasites carrying the vaccine allele [8]. Collectively, these results point out the need to study *P. falciparum* polymorphism as an important step toward the identification of efficient malaria vaccine candidates.

The study presented here falls within the framework of preliminary investigations aimed at establishing a malaria clinical trial site in the district of Toubacouta (Fatick region, Senegal). It follows epidemiological [9], entomological [10] and immunological investigations undertaken in the same area. The objective is to characterize the genetic diversity of *P. falciparum* isolates from asymptomatic children in the selected study site by PCR-amplification of polymorphic regions of the two marker genes *msp*-*1* and *msp*-*2*. The generated data are used to analyse the potential relationship between the levels of parasitaemia and specific parasite’s allelic types.

## Methods

### Study area

**Fig. 1:**
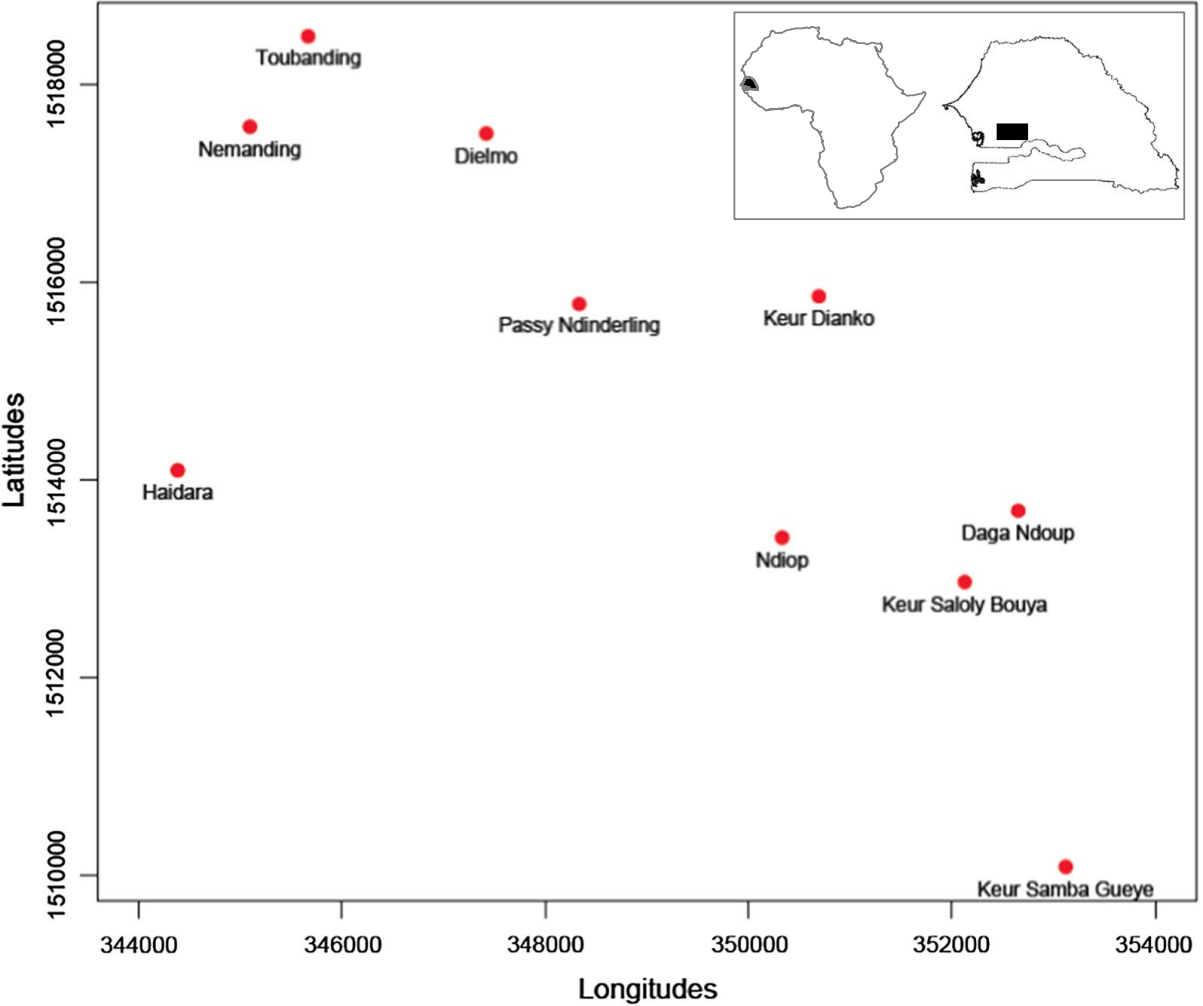
Map of the district of Toubacouta in Senegal. The eight villages of the study are shown as well as the Dielmo and Ndiop research centers located nearby

The study was conducted using samples from the rural community of Toubacouta situated in the region of Fatick (central-southern Senegal). The study area included eight villages: Haïdara, Daga Ndoup, Keur Ndianko, Nemanding, Passy Ndinderling, Keur Saloly Bouya, Keur Samba Gueye and Toubanding (Fig. 1). Malaria is mesoendemic in Toubacouta area with seasonal transmission occurring mainly during the rainy season between August and October, and with a peak in September [10]. The main vector belongs to the *Anopheles gambiae* complex. Malaria is mainly caused by *P. falciparum* with an entomological inoculation rate (EIR) of four infective bites per person from July to December 2011 in seven of the eight villages. Toubanding, the eighth village had an EIR of 30 infective bites per person owing to its proximity to the river Nema [10]. The population was estimated to 8000 inhabitants with 1500 children under 10 years (18.75%). Insecticide-treated nets were used by 52% of the population [10] and ACT was freely available to children.

### Sample collection

A total of 1316 children under 10 years of age were enrolled in the study from November to December 2010. Children were examined by a medical doctor and clinical parameters were properly recorded. A venous blood sample was taken and used for blood typing, measurement of biological parameters, and for preparation of thick and thin blood smears for malaria diagnosis. A symptomatic case of malaria was defined as an axillary temperature ≥ 37.5 °C or any reported history of fever within the last 24 h confirmed by RDT [9]. These cases were excluded from our study. Plasma and blood pellets were separated and stored at − 20 °C for serological and molecular biology analyses, respectively.

### Microscopic examination

Thick and thin smears were stained with 10% Giemsa for 25 min and microscopically examined for determination of parasite density and identification of species and developmental stages respectively. The number of parasites per 200 white blood cells (WBC) in thick-film was recorded and parasite density was estimated by counting the number of leucocytes by field examined and by arbitrarily considering that 8000 leucocytes were present in 1 µl of blood. At least 200 thick-film fields were examined before a slide was declared negative. Two experienced microscopists read the blood smears and in the case of a discrepancy, a third microscopist examined the slide. A slide was considered positive after two concordant readings by two different microscopists. Parasitaemias were classified into five levels including F1 (< 50 parasites/µl blood), F2 (50–499 parasites/µl), F3 (500–4999 parasites/µl), F4 (5000–49,999 parasites/µl) and F5 (≥ 50,000 parasites/µl) [11].

### Extraction of parasite DNA

Genomic DNA (gDNA) was extracted from 100 μl packed red blood cells using QiaAmp DNA mini kit (Qiagen, Hilden, Germany) according to the manufacturer’s instructions. The DNA was recovered in 50 μl of elution buffer provided with the kit into a properly labelled Eppendorf tube. DNA concentration and purity were estimated using a NanoDrop Lite Spectrophotometer (Thermo Scientific). Extracted DNAs were stored at − 20 °C until used.

### Genotyping of *Plasmodium falciparum* isolates

Genotyping of *P. falciparum* isolates was performed using a nested PCR approach targeting *msp*-*1* and *msp*-*2*, two highly polymorphic region of the parasite’s genome as previously described [12]. All primers used were synthesized from Tib Molbiol (Germany). In the primary reaction, the primers used span the entire *msp*-*1* block 2 and *msp*-*2* block 3 respectively. The initial amplification was followed by individual nested PCR reactions using primers specific for K1, MAD20, and RO33 allelic types of *msp*-*1*, and FC27 and 3D7 allelic types of *msp*-*2*. In details, in the first PCR, 2.0 μl of DNA was amplified with 12.5 μl of 2× GMM (Go Taq Green Master Mix, Catalogue no M7113 Promega), 1 μl of forward primer (10 μM), 1 μl of reverse primer (10 μM) and sterile ultrapure water to a final volume of 25 μl. For the second round of PCR, 2.0 μl of first-round PCR products was amplified with 12.5 μl of 2× GMM (Go Taq Green Master Mix, M7113 Promega), 1.0 μl of forward primer (10 μM), 1.0 μl of reverse primer (10 μM) and sterile ultrapure water to a final volume of 25 μl. Primers and the reaction conditions are shown in Additional file 1: Table S1. Positive and negative controls were included in each set of reactions. Ten microliters of each PCR product were separated by electrophoresis on a 1.5% agarose gel that was stained with ethidium bromide. The size of the PCR products was estimated using a DNA ladder (1 kb Plus DNA ladder, Invitrogen). Amplified DNA was visualized by ultraviolet trans-illumination using an E-GEL IMAGER (Life Technologies). Gel photographs (Additional file 2: Fig. S1) were re-scored by visual comparison of DNA fragments and genotypes were identified according to band sizes for each individual sample. The size polymorphism for each allelic family was analysed assuming that one band represented one amplified PCR fragment derived from a single copy of *P. falciparum msp*-*1* or *msp*-*2* gene.

### Statistical analysis

Asymptomatic malaria infection was defined as the presence of parasitaemia in the absence of symptoms or treatment. The complexity of infection (COI) also referred as multiplicity of infection (MOI) represented by the number of fragments per infected person, is the average number, for each gene, of distinct fragments per PCR positive sample. Expected heterozygosity (H_E_) was defined as the probability of being infected by two parasites with different alleles at a given locus. It is ranged between 0 and 1 was calculated by using the following formula: ${\text{H}}_{\text{E}} \, = \,\left[ {{\text{n}}/\left( {{\text{n}} - 1} \right)} \right] \, \left[ {\left( { 1- \sum {\text{pi2}}} \right)} \right]$, where n is the number of isolates sampled and pi is the allele frequency at a given locus [13]. Allelic frequency was estimated by calculating the percentage of fragments assigned to one family or family combination out of the total number of alleles detected for each gene [14]. Multiple infections (MI) or polyclonal infections corresponded to the proportion of isolates with more than one amplified PCR fragment. Comparison between mean parasite density of monoclonal and polyclonal infection was performed using the Student T test. Association between parasitaemia and allele frequencies was estimated using the Pearson correlation method. Differences were considered significant when a *P* value was < 0.05. All analyses were performed using R software (version 3.0.2).

## Results

### Demographic and parasitological data

Of the 1316 samples analysed by microscopic observation, 91 (6.91%) were positive for *P. falciparum* while no other *Plasmodium* species were detected. Four positive samples were excluded from the study due to the presence of fever at the time of blood collection. The mean age for the remaining 87 asymptomatic children was 6.5 years and the sex ratio (F/M) was 0.74 (Table 1). The mean parasite density was 8915.7 parasites/μl of blood, while the median was 960 parasites/μl with a range from 0 to 106,560 parasites/μl. These parasitaemias only referred to the amount of asexual blood stage parasites. Gametocytes presence was reported without quantification. Hence, a sample was considered positive with 0% asexual blood stage parasites if gametocyte presence was confirmed [15]. The genotyping success rate was 96.5% (84/87) and 94% (82/87) for *msp*-*1* and *msp*-*2*, respectively.

**Table 1:** Demographic and parasitological characteristics of *Plasmodium falciparum* positive asymptomatic children (N = 87)

Characteristics of patients	Values
Mean age in years (± SD)	6.5 (± 2.2)
Median age (range)	6.8 (0.9 to 9.9 years)
Sex ratio (F/M)	0.74 (37/50)
Mean of parasite density (parasites/µl)	8915.7
Median of parasite density (range)	640 (0 to 106,560)

### Allelic diversity of *Plasmodium falciparum msp*-*1* and *msp*-*2* genes

For *msp*-*1* gene, 15 different alleles were identified including 6, 5 and 4 belonging to the K1, MAD20 and RO33 allelic families respectively. The total number of *msp*-*1* alleles detected in all samples was 219. Allele frequencies were 40.2% (88/219), 31.1% (68/219), and 28.7% (63/219) for RO33, MAD20 and K1, respectively (Table 2). Expected heterozygosity (H_E_) was 0.67 for *msp*-*1* loci. Of *msp*-*1* positive samples, 69% (58/84) were polyclonal. The mean complexity of infection (COI) was 2.5. These polyclonal infections mostly involved trimorphic and dimorphic allelic family combinations: 27.4% (23/84) of K1/MAD20/RO33, 16.7% (14/84) of MAD20/RO33, 14.3% (12/84) of K1/RO33 and 5.9% (5/84) of K1/MAD20. The maximum number of genotypes belonging to the same allelic family detected in a single isolate was 3, 5, and 4, for K1, MAD20 and RO33, respectively.

**Table 2:** Allele families and diversity of *Plasmodium falciparum* genetic profiles

Allelic type	N (%)	Allelic type	N (%)
*msp-1*		*msp-2*	
K1	12 (14.3)	3D7	20 (24.4)
MAD20	8 (9.5)	FC27	10 (12.2)
RO33	10 (11.9)	3D7/FC27	52 (63.4)
K1/MAD20	5 (5.9)		
K1/RO33	12 (14.3)		
MAD20/RO33	14 (16.7)		
K1/MAD20/RO33	23 (27.4)		
Total combinations	84 (100)		82 (100)
Total K1	63 (28.7)	Total 3D7	206 (53.7)
Total MAD20	68 (31.1)	Total FC27	178 (46.3)
Total RO33	88 (40.2)		
Total alleles	219		384
MI	58 (69)		73 (89)
COI	2.5		4.7

MI: prevalence of multiples infections; COI: complexity of infection

For *msp*-*2* gene, 21 different alleles were detected with 10 for 3D7 allelic family and 11 for FC27. Allelic family frequencies showed that the 3D7 allelic type was predominant (53.7%, 206/384) over FC27 (46.3%, 178/384) (Table 2). The prevalence of multiple infections (MI) was 89% (73/82). Expected heterozygosity (H_E_) was 0.50 for *msp*-*2* loci. The prevalence of multiple infections (MI) was 89% (73/82). The mean complexity of infection was 4.7. The frequency of infections involving the two allelic families of *msp*-*2* was 63% (52/82). The maximum number of genotypes to belong to the same allelic family detected in a single isolate was 5 and 7 for 3D7 and FC27, respectively.

Ranges of fragment sizes for *msp*-*1* and *msp*-*2* allelic families are shown in Additional file 3: Table S2.

### Parasitaemia is higher in polyclonal than in monoclonal asymptomatic infection by *Plasmodium falciparum*

**Fig. 2:**
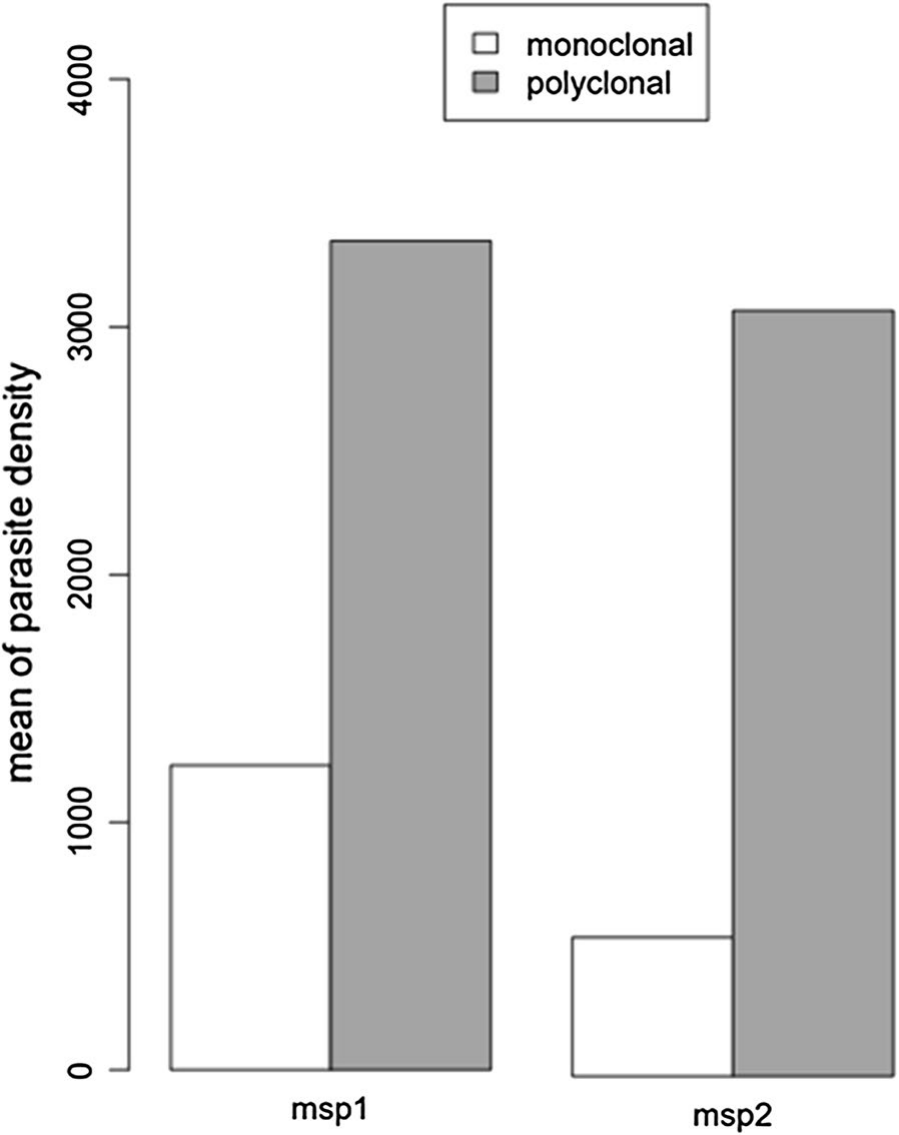
Comparison of mean parasite density between polyclonal and monoclonal infection. Parasite density is higher in *Plasmodium falciparum* polyclonal than in monoclonal infection in asymptomatic children when analysed with both *msp*-*1* and *msp*-*2* genotyping

Parasitaemias were compared in samples with polyclonal versus monoclonal infections. The analyses were performed after removal of eight samples that were found to be outliers (extreme 5% values showed as dots of boxplot in Additional file 4: Fig S2). Mean parasite density was significantly higher (Student T test, P = 0.012) in polyclonal (mean density = 3347 parasites/μl) than in monoclonal infection (mean density of 1229 parasites/μl) for *msp*-*1* positives isolates (Fig. 2). A similar result was observed with *msp*-*2* (Fig. 2), with significantly higher parasitaemia (Student T test, P = 0.00008) in polyclonal (mean density of 3092 parasites/μl) than in monoclonal infections (561 parasites/μl).

### *msp*-1 and *msp*-2 allelic families influence parasitaemia in asymptomatic *Plasmodium falciparum* infection

**Fig. 3:**
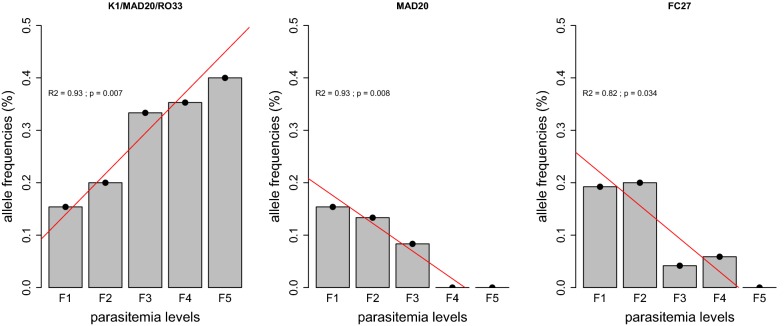
Correlation between frequency of unique or allelic family combinations and levels of parasitaemia. F1 (< 50 parasites/µl blood), F2 (50–499 parasites/µl), F3 (500–4999 parasites/µl), F4 (5000–49,999 parasites/µl) and F5 (≥ 50,000 parasites/µl)

To further analyse the effect of polyclonal infection on parasitaemia, the influence of specific allele combinations on parasite density was assessed. For this purpose, the samples were classified according to their parasite density and five levels of parasitaemia were set. Analysis of the relationship between *msp*-*1* allelic family combinations and parasitaemia (Fig. 3) showed a significant positive association between trimorphic infections K1/MAD20/RO33 and parasitaemia (r^2^ = 0.93; P = 0.007). In contrast, parasitaemia were significantly and negatively correlated with MAD20 monoallelic infections (r^2^ = 0.93; P = 0.008). Regarding *msp*-*2,* FC27 monoallelic infections were negatively associated with parasitaemia (r^2^ = 0.82; P = 0.034).

## Discussion

Preliminary studies for a clinical trial site for malaria have been undertaken in the district of Toubacouta (Fatick region, Senegal). Eight villages were involved. The genetic diversity of *P. falciparum*, the only species diagnosed, was analysed in samples from asymptomatic children collected through a cross-sectional survey. An important allelic diversity was observed with 15 and 21 different alleles, and 69% and 89% of multiple infections, for *msp*-*1* and *msp*-*2* genes, respectively. The high genetic diversity in our study is in agreement with previous findings on asymptomatic children in the mesoendemic malaria site of Niakhar in Senegal. In Niakhar, genotyping with the *msp*-*2* polymorphic marker revealed a range from 2 to 7 different fragments per carrier with 64% of multiclonal infections [16]. Similar results were observed 16 years ago in Ndiop in asymptomatic children [14]. Comparison of genetic diversity of *P. falciparum* isolates in Ndiop (1994) [14] and our study area (2010) in the nearby locality shows a stable genetic diversity over time. A high level of diversity persists despite a 12 times difference in transmission levels (63 infected bites per person per 4 months in 1994 compared to four infected bites per person per 3 months in 2010) between the two periods as a result of malaria control interventions [17]. This observation underlines that *P. falciparum* genetic diversity does not only rely on parasite transmission rate. Thus, it has been demonstrated that the genotypic profile changes within hours or days in asymptomatic infections [18].

Unlike genetic diversity, complexity of infection (COI) is more commonly associated with the level of malaria transmission. In line with this observation, Konate et al. [19] found that COI was twice higher in the holoendemic area of Dielmo than in the mesoendemic village of Ndiop. Low COI was also associated with low transmission rate in Malaysia [13]. Vafa et al. [16] highlighted COI variation within the same year according to transmission period. In this study, COI is consistent with results from mesoendemic regions for both *msp*-*1* and *msp*-*2* markers [14, 20, 21].

Association between the number of clones per sample and the level of parasitaemia has been investigated. The results show that polyclonality is likely to be associated with high parasitaemia. This is consistent with previous studies [16, 21–23]. On the one hand, parasite density is attributed a major role in malaria physiopathology and some authors suggest that it might be used as a marker for morbidity and mortality associated with this disease [24, 25]. On the other hand, it is considered as the centerpiece of malaria immunity. In this regard, it has been shown that asymptomatic polyclonal carriage may protect against clinical malaria [26, 27]. Although the mechanism of this partial protection remains unclear, it is probably by maintaining protective immune responses to asexual blood stage parasites [21, 28, 29].

Moreover, this study emphasizes the significant positive correlation between frequencies of trimorphic infections K1/MAD20/RO33 of *msp*-*1* gene and parasitaemia (Fig. 3). Although the representative alleles of this combination have to be identified, these preliminary results point out the necessity of multivalent vaccine candidates to overcome antigenic diversity [30].

This study has some technical limitations. Firstly, PCR cannot discriminate between alleles of differing sequences with similar size and can thus underestimate the number of distinct alleles [30, 31]. Secondly, polymorphism of sub-microscopic infections is not taken into account in this study. Despite these limitations, the results provide an insight on the genetic diversity of *P. falciparum* clones circulating in Toubacouta, Senegal.

## Conclusions

Despite the general decline in malaria prevalence observed following the implementation of various malaria control strategies, *P. falciparum* strains are substantially circulating in this study site and display a high genetic diversity. The level of this diversity is comparable to that found in malaria endemic and mesoendemic areas, therefore, making the site interesting for potential vaccine and therapeutic clinical trials. The positive correlation between *msp*-*1* trimorphic infection and parasitaemia suggests the use of field information on genetic diversity as starting blocks for designing new malaria vaccines.

## Additional files

**Additional file 1: Table S1.** Primers and the reaction conditions used to amplify *msp-1* and *msp-2* genes.

**Additional file 2: Fig. S1.** Photographs of migration profiles of *msp-1* and *msp-2* allelic families in 1.5% agarose gel.

**Additional file 3: Table S2.** Ranges of fragment sizes for *msp-1* and *msp-2* allelic families.

**Additional file 4: Fig. S2.** Box plot of parasitaemia in samples from asymptomatic children infected with *Plasmodium falciparum*. The eight outliers removed prior to comparison of monoclonal and polyclonal infections are shown.

## Abbreviations

RDT: rapid diagnostic test
IRS: indoor residual spraying
LLIN: long-lasting insecticide-treated nets
ACT: artemisinin-based combination therapy
IPTp: intermittent preventive treatment in pregnancy
SMC: seasonal malaria chemoprevention
*msp1*: merozoite surface protein 1
*msp2*: merozoite surface protein 2
EIR: entomological inoculation rate
DNA: deoxyribonucleic acid
PCR: polymerase chain reaction
UV: ultraviolet
COI: complexity of infection
MOI: multiplicity of infection
MI: multiple infections
bp: base pairs
H_E_: expected heterozygosity
