# Supplementary material for: Association of high Plasmodium falciparum parasite densities with polyclonal microscopic infections in asymptomatic children from Toubacouta, Senegal
Source: Malar J. 2019 Feb 21;18:48. doi: 10.1186/s12936-019-2684-3 (PMC6385392; doi:10.1186/s12936-019-2684-3)
Supplement: Supplementary file 1 — Additional file 1: Table S1. Primers and the reaction conditions used to amplify msp-1 and msp-2 genes. [file 12936_2019_2684_MOESM1_ESM.docx]

| **Primary PCR** |  | **primer** | **Primer sequence** | **Allelic family** |
| --- | --- | --- | --- | --- |
|  | ***msp-1*** | M1-OF | 5’CTAGAAGCTTTAGAAGATGCAGTATTG 3’ |  |
|  |  | M1-OR | 5’CTTAAATAGTATTCTAATTCAAGTGGATCA3’ |  |
|  |  |  | **Cycling conditions** |  |
|  |  | Initial denaturation: 94°C for 2 min; PCR: 30 cycles of 94°C for 30 s, 54°C for 1 mn, 72°C for 1 mn; final elongation of 72°C for 5 mn; hold at 15°C | |  |
|  | ***msp-2*** | M2-OF | 5’ATGAAGGTAATTAAAACATTGTCTATT ATA3’ |  |
|  |  | M2-OF | 5’ATGAAGGTAATTAAAACATTGTCTATT ATA3’ |  |
|  |  |  | **Cycling conditions** |  |
|  |  | Initial denaturation: 94°C for 5 min; PCR: 30 cycles of 94°C for 1 mns, 60°C for 1 mn, 72°C for 2 mn; final elongation of 72°C for 5 mn; hold at 15°C | |  |
| **Secondary PCR** | ***msp-1*** | M1-KF | 5’AAATAGAGAAGAAATTACTACAAAAGGTGC3’ | **K1** |
|  |  | M1-KR | 5’GCTTGCATCAGCTGGAGGGCTTGCACCAGA3’ |  |
|  |  | M1-MF | 5’AAATGAAGGAACAAGTGGAACAGCTGTTAC3’ | **MAD20** |
|  |  | M1-MR | 5’ATCTGAAGGATTTGTACGTCTTGAATTACC3’ |  |
|  |  | M1-RF | 5’TAAAGGATGGAGCAAATACTCAAGTTGTTG3’ | **RO33** |
|  |  | MI-RR | 5’CAAGTAATTTTGAACTCTATGTTTTAAATCAGCGTA3’ |  |
|  |  |  | **Cycling conditions** |  |
|  |  |  | Initial denaturation: 94°C for 2 min; PCR: 30 cycles of 94°C for 30 s, 59°C for 1 mn, 72°C for 1 mn; final elongation of 72°C for 5 mn; hold at 15°C |  |
|  | ***msp-2*** | M2-1CF | 5’GCAGAAAGTAAGCCTTCTACTGGTGCT3’ | **3D7** |
|  |  | M2-1CR | 5’CTGAAGAGGTACTGGTACTGGTAG3’ |  |
|  |  | M2-FCF | 5’GCAAATGAAGGTTCTAATACTAATAG3’ | **FC27** |
|  |  | M2-FCR | 5’GCATTGCCAGAATTGAA3’ |  |
|  |  |  | **Cycling conditions** |  |
|  |  |  | Initial denaturation: 94°C for 5 min; PCR: 30 cycles of 94°C for 1 mns, 60°C for 1 mn, 72°C for 2 mn; final elongation of 72°C for 5 mn; hold at 15°C |  |

**Table S1 Primers and the reaction conditions used to amplify *msp-1* and *msp-2* genes**
