# Supplementary material for: Association of high Plasmodium falciparum parasite densities with polyclonal microscopic infections in asymptomatic children from Toubacouta, Senegal
Source: Malar J. 2019 Feb 21;18:48. doi: 10.1186/s12936-019-2684-3 (PMC6385392; doi:10.1186/s12936-019-2684-3)
Supplement: Supplementary file 2 — Additional file 2: Fig. S1. Photographs of migration profiles of msp-1 and msp-2 allelic families in 1.5% agarose gel. [file 12936_2019_2684_MOESM2_ESM.pdf]

S S S S S S S S S N P PM

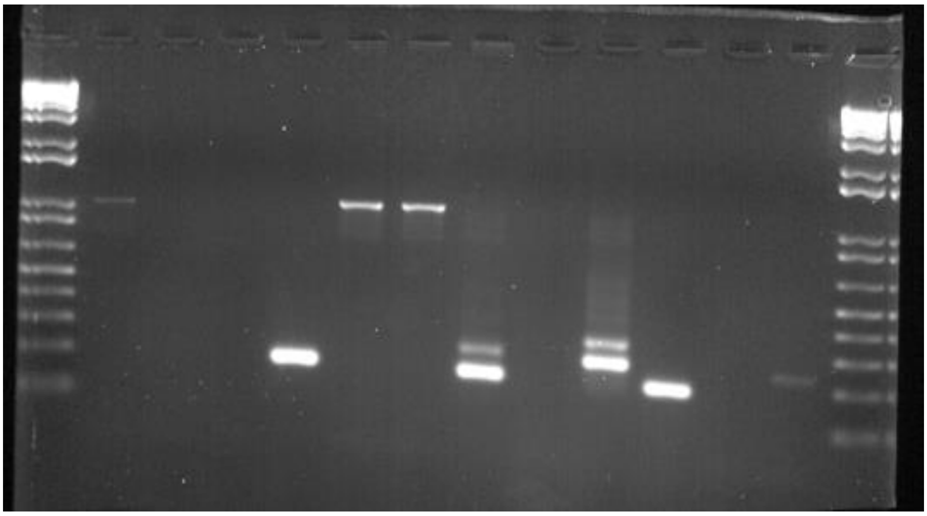

K1 allelic family (*msp-1)*

PM: DNA ladder (HyperLadder 1kb 100 lanes, Bioline); P: positive control; N: negative control, S: sample

S S S S S S S S S N P PM

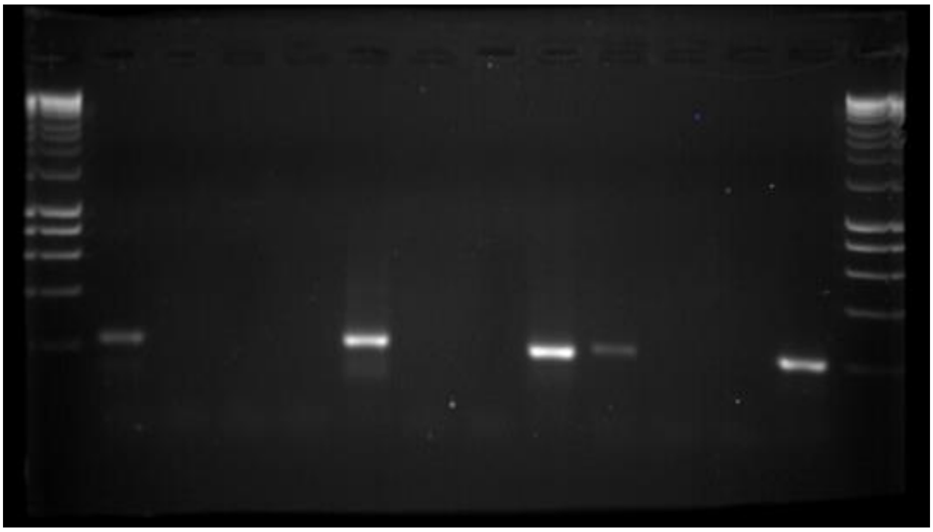

MAD20 allelic family (*msp-1)*

PM: DNA ladder (HyperLadder 1kb 100 lanes, Bioline); P: positive control; N: negative control, S: sample

S S S S S S S S S N P PM

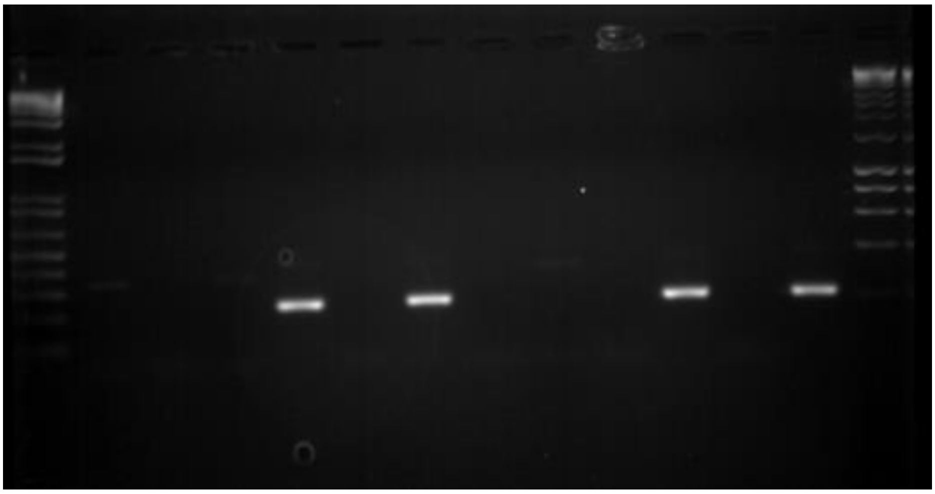

RO33 allelic family (*msp-1)*

PM: DNA ladder (HyperLadder 1kb 100 lanes, Bioline); P: positive control; N: negative control, S: sample

S S S S S S S S S N P PM

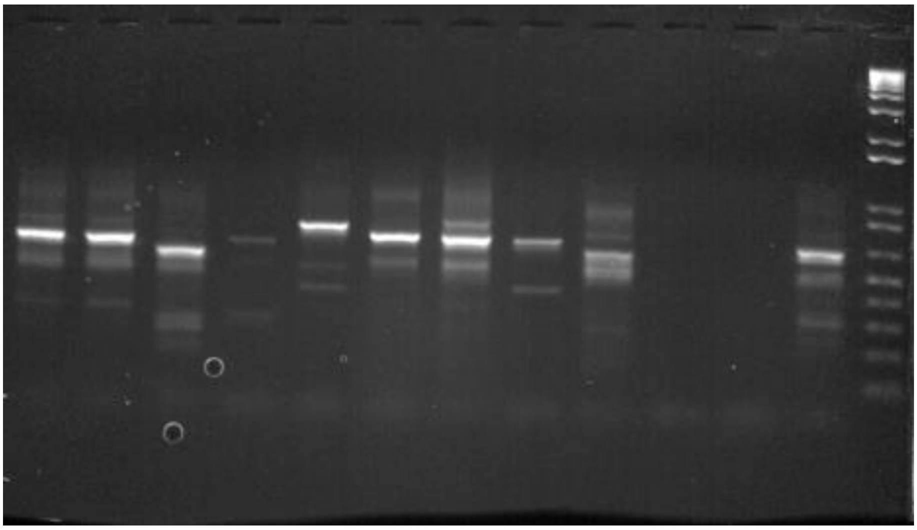

3D7 allelic family (*msp-2)*

PM: DNA ladder (HyperLadder 1kb 100 lanes, Bioline); P: positive control; N: negative control, S: sample

S S S S S S S S S S N P PM

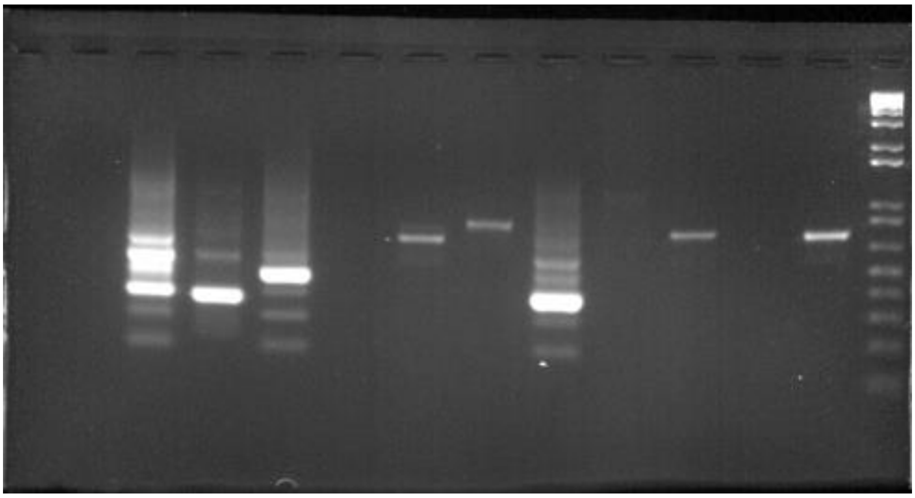

FC27 3D7 allelic family (*msp-2)*

PM: DNA ladder (HyperLadder 1kb 100 lanes, Bioline); P: positive control; N: negative control, S: sample
