# Supplementary material for: Association of high Plasmodium falciparum parasite densities with polyclonal microscopic infections in asymptomatic children from Toubacouta, Senegal
Source: Malar J. 2019 Feb 21;18:48. doi: 10.1186/s12936-019-2684-3 (PMC6385392; doi:10.1186/s12936-019-2684-3)
Supplement: Supplementary file 3 — Additional file 3: Table S2. Ranges of fragment sizes for msp-1 and msp-2 allelic families. [file 12936_2019_2684_MOESM3_ESM.docx]

|  | Base pair range | | | | | | |
| --- | --- | --- | --- | --- | --- | --- | --- |
|  | **100-200bp** | **300-400bp** | **500-700bp** | **100-200bp** | | **300-400bp** | **500-700bp** |
| *msp-1* | Single fragment **N**(%) | | | | Multiple fragments **N**(%) | | |
| RO33  N=88 | **6**(14,29) | **33**(85,71) | 0 | **36**(72,55) | | **7**(15,03) | **6**(12,42) |
| MAD20  N=68 | **29**(73,33) | 0 | **10**(26,67) | **13**(45,45) | | **5**(18,18) | **11**(36,36) |
| K1  N=63 | **1**(2,56) | **32**(76,92) | **9**(20,51) | **5**(25,00) | | **15**(70,00) | **1**(5,00) |
| *msp-2* |  |  |  |  | |  |  |
| FC27  N=178 | **1**(2,94) | **1**(2,94) | **23**(94,12) | **48**(31,25) | | **87**(56,25) | **19**(12,50) |
| 3D7  N=206 | 0 | **9**(56,25) | **7**(43,75) | **28**(14,67) | | **68**(36,00) | **94**(49,33) |

**Table S2 Ranges of fragment sizes for *msp-1* and *msp-2* allelic families**
