# Supplementary figures and images for: Association of high Plasmodium falciparum parasite densities with polyclonal microscopic infections in asymptomatic children from Toubacouta, Senegal
Source: Malar J. 2019 Feb 21;18:48. doi: 10.1186/s12936-019-2684-3 (PMC6385392; doi:10.1186/s12936-019-2684-3)

Parasitemia's boxplot

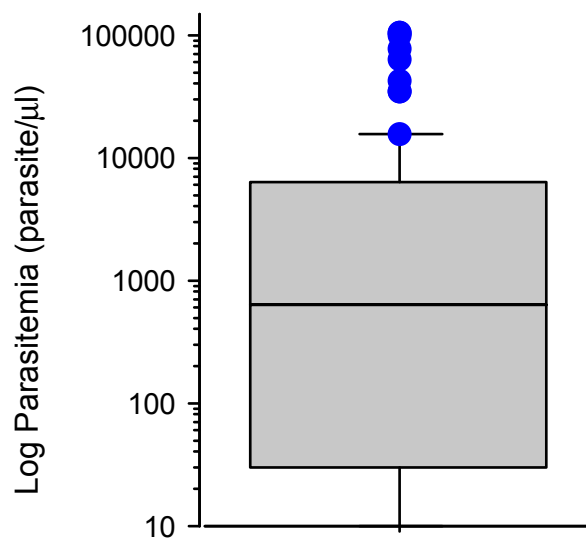

Supplement: Supplementary file 4 — Additional file 4: Fig. S2. Box plot of parasitaemia in samples from asymptomatic children infected with Plasmodium falciparum. The eight outliers removed prior to comparison of monoclonal and polyclonal infections are shown. [file 12936_2019_2684_MOESM4_ESM.pdf]
